# Longitudinal association between hemoglobin and lung function with insights into the incidence of airflow obstruction: an observational study

**DOI:** 10.1186/s12890-025-03505-3

**Published:** 2025-01-30

**Authors:** Jihoon Kim, Yun Tae Kim, Ah Young Leem, Ji Ye Jung, Young Sam Kim, Youngmok Park

**Affiliations:** 1 Department of Internal Medicine, Severance Hospital, Yonsei University College of Medicine, Seoul, Republic of Korea; 2 Division of Biostatistics, Kangbuk Samsung Hospital, Seoul, Republic of Korea; 3 Division of Pulmonary and Critical Care Medicine, Department of Internal Medicine, Severance Hospital, Yonsei University College of Medicine, Seoul, Republic of Korea; 4 Institute for Innovation in Digital Healthcare, Yonsei University, 50-1 Yonsei-ro, Seodaemun–gu, Seoul, 03722 Republic of Korea

**Keywords:** Airflow obstruction, Hemoglobin, Spirometry

## Abstract

**Background/Aims:**

Evidence regarding the long-term association between hemoglobin (Hb) levels and lung function in individuals from the general population is scarce. This study aimed to determine the longitudinal association between Hb levels and lung function in a community-based population cohort in South Korea.

**Methods:**

We used linear mixed regression analysis to evaluate the longitudinal associations between Hb levels and lung function parameters, including forced vital capacity (FVC), forced expiratory volume in 1 s (FEV_1_), and FEV_1_/FVC. Additionally, we used a generalized estimating equation to calculate the odds ratio (OR) of airflow obstruction (AO) according to the Hb level.

**Results:**

Over an 8-year biennial follow-up of 4,468 individuals (median age, 53.9 years; men, 49.0%), we observed that in men, Hb levels were positively associated with lung function (estimated values of FVC: 16.7 mL, FEV_1_: 15.5 mL, FEV_1_/FVC: 0.18%; all *P* < 0.001) and a decreased incidence of AO (OR = 0.83, *P* < 0.001). In women, Hb levels were positively associated with FVC but not with FEV_1_ or FEV_1_/FVC (estimated values of FVC: 4.7 mL, *P* = 0.045; FEV_1_: 3.1 mL, *P* = 0.142; FEV_1_/FVC: 0.01%, *P* = 0.838). The incidence of AO was not significantly different among women (OR = 0.93, *P* = 0.568). In postmenopausal women, higher Hb levels were associated with increased lung function (estimated values of FVC: 11.8 mL, *P* < 0.001; FEV_1_: 9.8 mL, *P* < 0.001; FEV_1_/FVC: 0.09%, *P* = 0.052), but the incidence of AO was not statistically significant (OR = 0.82, *P* = 0.129).

**Conclusions:**

A decreased Hb level was associated with reduced lung function and an increased incidence of AO in men.

**Supplementary Information:**

The online version contains supplementary material available at 10.1186/s12890-025-03505-3.

## Key messages

1. A decreased hemoglobin level was associated with a decline in spirometry results regardless of gender.

2. A lower hemoglobin level was associated with a higher incidence of airflow obstruction in men.

3. A decreased hemoglobin level should raise awareness about impaired lung function.

**Supplementary Information:**

The online version contains supplementary material available at 10.1186/s12890-025-03505-3.

## Introduction

Chronic obstructive pulmonary disease (COPD) is a major cause of mortality and morbidity worldwide, resulting in significant healthcare burden and societal costs [1–3]. COPD accounts for the highest proportion of mortality among all respiratory diseases and ranks third in Europe [4, 5] and fourth in the United States as the leading cause of death [6, 7]. Comorbidities such as cardiovascular disease, osteoporosis, gastroesophageal reflux, and depression are commonly reported in patients with COPD. Recently, anemia has been recognized as a frequent comorbidity of COPD [8].

In the past, patients with COPD were often exposed to hypoxemia, resulting in a higher prevalence of secondary polycythemia [9]. Recently, the prevalence of polycythemia in COPD has decreased owing to the prevention of hypoxemia through long-term oxygen therapy [10]. In contrast, recent studies have reported that anemia is significantly associated with mortality, quality of life, and hospitalization rates in patients with COPD [11–15]. The mechanisms underlying anemia in patients with COPD are multifactorial and have not been conclusively elucidated [16].

Several studies have reported an association between hemoglobin (Hb) levels and lung function; however, most were cross-sectional and focused solely on patients with COPD, excluding healthy individuals [11, 13–15]. In this study, we investigated the longitudinal association between Hb levels and lung function in individuals from the general population in South Korea. We also explored the effects of anemia on the incidence of COPD.

## Patients and methods

### Study population

**Fig. 1:**
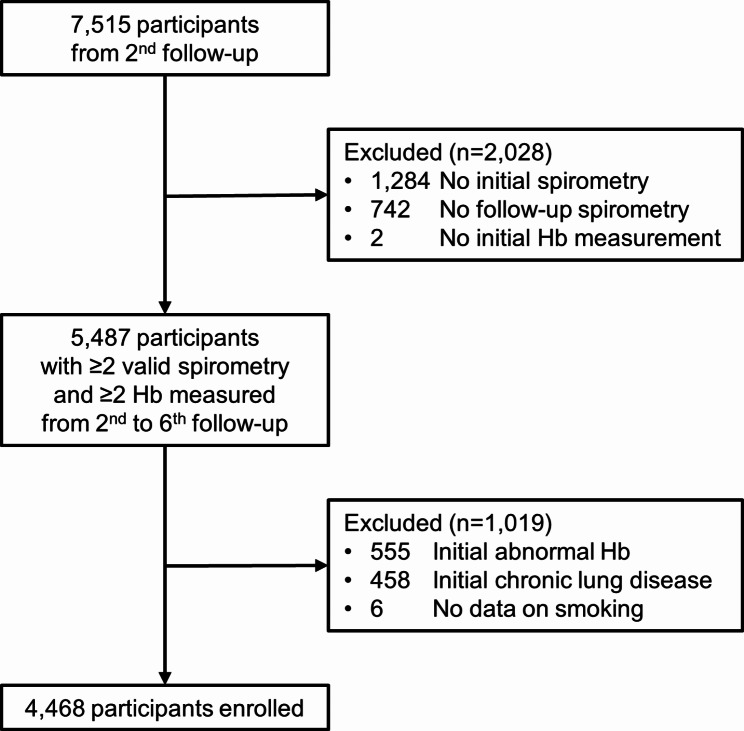
Flowchart of the participant selection process. Chronic lung disease refers to either baseline FEV_1_/FVC < 70% or current inhaler use. Abnormal hemoglobin level indicates anemia or erythrocytosis Abbreviations: FEV_1_, forced expiratory volume in 1 s; FVC, forced vital capacity; Hb, hemoglobin

We recruited participants from the Ansan–Ansung cohort, which is part of the National Genome Research Institute-supported Korean Genome and Epidemiology Study [17, 18]. The cohort is population-based, involving South Koreans aged 40–69 years from two distinct locations, including Ansan, a 555,000-person urban community, and Ansung, a 133,000-person rural community [18]. Participants were enrolled from 2001 to 2002 and followed up every 2 years until the 6th follow-up (2013–2014). The number of participants at each follow-up visit is presented in Table S1. Since quality-controlled spirometry results were obtained at the second follow-up, we included 7,515 participants from the second follow-up in our study as the initial analysis point. The following participants were excluded: (1) those without initial spirometry data in the second follow-up; (2) those without follow-up spirometry data at least once during the 3rd − 6th follow-up; (3) those without Hb data in the second follow-up; (4) those with chronic lung disease (forced expiratory volume in 1 s [FEV_1_]/forced vital capacity [FVC] < 70% or current inhaler use); (5) those who met the anemia [19] or erythrocytosis criteria [20] of the World Health Organization (WHO); (6) those without smoking data in the second follow-up. Consequently, 4,468 participants who met the inclusion criteria were enrolled in the study (Fig. 1).

The Korea Centers for Disease Control and Prevention obtained written informed consent from all participants regarding the collection of their data. The Institutional Review Board of Severance Hospital approved the study protocol (4-2022-0558). All methods were performed per the approved protocol and the relevant guidelines and regulations.

### Spirometry

At baseline and at every follow-up visit, lung function was evaluated using spirometry (Vmax2130, Sensor-Medics, Yorba Linda, CA). All tests were conducted according to the standardized protocols of the American Thoracic Society, and the Korean equation was used as a reference for normal lung function [21, 22].

### Airflow obstruction

According to the Global Initiative for Chronic Obstructive Pulmonary Disease, we diagnosed airflow obstruction (AO) when the FEV_1_/FVC was < 70% on spirometry [23]. Once a patient was diagnosed with AO, they were consistently classified as having AO, regardless of the subsequent spirometry results.

### Hemoglobin

We measured the Hb levels of participants at baseline and at every follow-up visit. We excluded patients with anemia or erythrocytosis at baseline to examine the effect of Hb levels on lung function in the general population. According to the WHO diagnostic criteria, anemia is defined as Hb levels < 13 g/dL in men and < 12 g/dL in women, whereas erythrocytosis is defined as Hb levels > 18.5 g/dL in men and > 16.5 g/dL in women [19, 20].

### Subgroup analyses: postmenopausal women

While Hb levels in menstruating women may fluctuate based on the timing of measurement [24], it was challenging to account for this factor using our available data. Therefore, we conducted a subgroup analysis including postmenopausal women to exclude the influence of menstruation-related Hb fluctuations.

### Statistical analyses

To compare the baseline characteristics between the two groups, we used Student’s t-test or the Mann–Whitney U test for continuous variables and the Pearson χ^2^ test and Fisher’s exact test for categorical variables. Because the baseline characteristics and definitions of erythrocytosis and anemia differ by gender [19, 20], we analyzed men and women separately.

We performed multiple linear regression analyses to evaluate the cross-sectional associations between baseline Hb levels and lung function parameters. In these analyses, lung function was represented by predicted values adjusted for age, height, and weight, rather than absolute values. Furthermore, the models were adjusted for smoking history, area of residence, and menopausal status. Notably, adjustments for pack-years smoked were exclusively made for men because of the disproportionately low proportion of women who smoked. Given this marginal representation, the inclusion of ever-smoker women in the analysis was considered impractical, and they were omitted.

We evaluated the longitudinal associations between lung function and Hb levels using multiple linear mixed regression analyses. For these analyses, absolute values of lung function parameters were used to assess changes over time, with adjustments made for age, height, smoking history, area of residence, and menopausal status. Baseline lung function was adjusted considering the effect of interpersonal variations on the magnitude of longitudinal decline [25]. Since we used participant identification number as a random effect in the multiple linear mixed regression analyses, the models were able to provide fixed effect estimates that reflect the individual differences in baseline levels and rates of change in lung function.

We also employed a generalized estimating equation (GEE) to determine whether the odds ratio (OR) for AO incidence varies with the Hb level. For GEE analysis, it is essential to have no missing values in the dataset. Therefore, we selected participants from the initial enrollment who had undergone spirometry and Hb measurements at least four out of five times. In cases with four measurements, missing values were substituted with the average of the nearest preceding and subsequent measurements. If the last measurement was missing, we estimated its value based on the trend observed in previous results. In addition, women with unavailable menopause data were excluded from the analysis.

Statistical analyses were performed using the R software version 4.0.2 (The R Foundation for Statistical Computing, Vienna, Austria). We used the lme4 package for multiple linear mixed regression analysis and the Gee package for the GEE. Statistical significance was set at two-tailed *P* < 0.05.

## Results

### Baseline characteristics

The baseline characteristics of the participants are presented in Table 1. The mean age was 53.9 ± 7.8 years (men: 52.9 ± 7.3 years, women: 54.9 ± 8.1 years), and 49% of the participants were men. The baseline body mass index was higher in women than in men (men: 24.6 kg/m^2^ vs. women: 24.9 kg/m^2^, *P* < 0.001), whereas men were taller than women (men: 167.4 cm vs. women: 154.3 cm, *P* < 0.001). The Hb level was also higher in men than in women (men: 15.1 g/dL vs. women: 13.1 g/dL, *P* < 0.001).

**Table 1:** Baseline characteristics and follow-up data of study participants

	Total (*N* = 4,468)	Men (*N* = 2,190)	Women (*N* = 2,278)	*P*-value
Age, years	53.9 ± 7.8	52.9 ± 7.3	54.9 ± 8.1	< 0.001
Height, cm	160.7 ± 8.6	167.4 ± 5.7	154.3 ± 5.4	< 0.001
Body mass index, kg/m^2^	24.8 ± 3.0	24.6 ± 2.7	24.9 ± 3.2	< 0.001
Smoking				< 0.001
Never	2805 (62.8)	582 (26.6)	2223 (97.6)	
Ex-smoker	888 (19.9)	869 (39.7)	19 (0.8)	
Current smoker	775 (17.4)	739 (33.7)	36 (1.6)	
Smoking exposure, pack-years	9.3 ± 16.1	18.7 ± 18.7	0.3 ± 2.8	
Residential area				< 0.001
Rural	1832 (41.0)	797 (36.4)	1035 (45.4)	
Urban	2636 (59.0)	1393 (63.6)	1243 (54.6)	
Lung function				
FVC, L	3.62 ± 0.85	4.27 ± 0.63	3.00 ± 0.50	< 0.001
FVC, % predicted	104.2 ± 12.4	101.9 ± 11.8	106.4 ± 12.6	< 0.001
FEV_1_, L	2.90 ± 0.66	3.37 ± 0.52	2.44 ± 0.42	< 0.001
FEV_1_, % predicted	112.5 ± 14.6	108.5 ± 13.1	116.5 ± 14.8	< 0.001
FEV_1_/FVC, %	80.2 ± 4.8	79.0 ± 4.8	81.4 ± 4.5	< 0.001
Hb level, g/dL	14.1 ± 1.3	15.1 ± 1.0	13.1 ± 0.7	< 0.001
Menopause			1541 (67.6)	
Measurements				
Spirometry, times	4 (3–5)	4 (3–5)	4 (3–5)	0.412
Hb, times	4 (3–5)	4 (3–5)	4 (3–5)	0.429
Follow-up duration, year	8 (6–8)	8 (6–8)	8 (8–8)	0.034

*Data are presented as numbers (%), means ± standard deviation, or median (interquartile range) unless otherwise indicated

Abbreviations: FEV_1_, forced expiratory volume in 1s; FVC, forced vital capacity; Hb, hemoglobin

The median follow-up period was 8 years (interquartile range, 6–8 years). During this period, lung function and Hb levels were measured for a median of four times (interquartile range, 3–5 times, respectively).

### Cross-sectional association between hemoglobin levels and lung function

The cross-sectional association between baseline Hb levels and lung function is shown in Table 2. In men, the Hb levels were positively associated with lung function parameters (FVC: estimate = 0.06%pred, FEV_1_: estimate = 0.06%pred, FEV_1_/FVC: estimate = 0.35%; all *P* < 0.001). However, in women, Hb levels were only positively associated with FEV_1_/FVC (estimate = 0.33%, *P* = 0.012).

**Table 2:** Multiple linear regression analysis of the cross-sectional associations between hemoglobin level and lung function in men, women, and postmenopausal women

	FVC, %pred			FEV_1_, %pred			FEV_1_/FVC, %		
	Estimate	Std Err	*P*-value	Estimate	Std Err	*P*-value	Estimate	Std Err	*P*-value
Men (*N* = 2,190)									
Hb level, g/dL	0.06	0.01	< 0.001	0.06	0.01	< 0.001	0.35	0.10	< 0.001
Smoking exposure, pack-years	0.00	0.00	0.012	0.00	0.00	0.701	-0.04	0.01	< 0.001
Area - Urban	0.11	0.02	< 0.001	0.15	0.02	< 0.001	0.77	0.21	< 0.001
Women (*N* = 2,223)									
Hb level, g/dL	-0.00	0.01	0.580	-0.01	0.01	0.224	0.33	0.13	0.012
Area - Urban	0.12	0.01	< 0.001	0.14	0.01	< 0.001	-0.27	0.20	0.168
Menopause - Yes	-0.22	0.01	< 0.001	-0.28	0.01	< 0.001	-1.47	0.21	< 0.001
Postmenopausal women (*N* = 1,507)									
Hb level, g/dL	0.00	0.01	0.835	-0.00	0.01	0.640	0.42	0.16	0.009
Area - Urban	0.15	0.01	< 0.001	0.16	0.01	< 0.001	-0.24	0.23	0.307

Abbreviations: FEV_1_, forced expiratory volume in 1s; FVC, forced vital capacity; Hb, hemoglobin

The estimate value indicates how much lung function parameters increase when the continuous variable increases by 1 unit, or when the categorical variable corresponds to the displayed value in the table, compared with the other value not shown

### Longitudinal association between hemoglobin levels and lung function

We examined the longitudinal association between Hb levels and lung function using multiple linear mixed regression (Table 3). In men, the Hb levels were positively associated with FVC, FEV_1_, and FEV_1_/FVC (FVC: estimate = 16.7 mL, FEV_1_: estimate = 15.5 mL, FEV_1_/FVC: estimate = 0.18%; all *P* < 0.001). However, in women, Hb levels were only positively associated with FVC (estimate = 4.7 mL, *P =* 0.045).

**Table 3:** Multiple linear mixed regression analysis of the long-term associations between hemoglobin level and lung function in men, women, and postmenopausal women

	FVC, mL			FEV_1_, mL			FEV_1_/FVC, %		
	Estimate	Std Err	*P*-value	Estimate	Std Err	*P*-value	Estimate	Std Err	*P*-value
Men (*N* = 2,190)									
Hb level, g/dL	16.7	2.4	< 0.001	15.5	2.2	< 0.001	0.18	0.03	< 0.001
Age, years	–2.2	0.5	< 0.001	–1.9	0.4	< 0.001	0.00	0.01	0.604
Height, cm	2.3	0.6	< 0.001	1.4	0.5	0.006	–	–	–
Smoking exposure, pack-years	–0.1	0.2	0.698	–0.1	0.1	0.293	0.00	0.00	0.234
Area - Urban	–97.6	6.3	< 0.001	–43.8	5.4	< 0.001	0.78	0.09	< 0.001
Baseline lung function†	928.0	6.0	< 0.001	919.3	6.4	< 0.001	0.93	0.01	< 0.001
Women (*N* = 2,223)									
Hb level, g/dL	4.7	2.4	0.045	3.1	2.1	0.142	0.01	0.04	0.838
Age, years	–1.6	0.4	< 0.001	–1.6	0.4	< 0.001	–0.02	0.01	< 0.001
Height, cm	2.9	0.5	< 0.001	1.5	0.4	< 0.001	–	–	–
Area - Urban	–82.3	4.9	< 0.001	–52.9	4.1	< 0.001	0.46	0.08	< 0.001
Menopause - Yes	–0.6	6.2	0.929	–3.1	5.3	0.550	–0.08	0.10	0.441
Baseline lung function†	900.6	6.5	< 0.001	900.9	6.6	< 0.001	0.91	0.01	< 0.001
Postmenopausal women (*N* = 1,507)									
Hb level, g/dL	11.8	2.9	< 0.001	9.8	2.6	< 0.001	0.09	0.05	0.052
Age, years	–1.7	0.4	< 0.001	–1.6	0.4	< 0.001	–0.02	0.01	0.009
Height, cm	2.9	0.7	< 0.001	1.5	0.5	0.006	–	–	–
Area - Urban	–83.5	5.9	< 0.001	–52.1	4.9	< 0.001	0.53	0.10	< 0.001
Baseline lung function†	899.4	8.1	< 0.001	899.7	8.3	< 0.001	0.91	0.01	< 0.001

Abbreviations: FEV_1_, forced expiratory volume in 1s; FVC, forced vital capacity; Hb, hemoglobin

The estimate value indicates how much lung function parameters increase when the continuous variable increases by 1 unit, or when the categorical variable corresponds to the displayed value in the table, compared with the other value not shown

†The values refer to the FVC, FEV1, and FEV1/FVC values measured at baseline

### Hemoglobin levels and the incidence of AO

Using a GEE, we examined how the OR for AO incidence varied with Hb levels. During the follow-up period, 282 of 1,594 men (17.7%) and 105 of 1,643 women (6.4%) were diagnosed with AO. As shown in Table 4, as the Hb level increased, the OR for AO incidence significantly decreased in men (OR = 0.83, *P* < 0.001), with no significant change observed in women (OR = 0.93, *P =* 0.568).

**Table 4:** Odds ratio for the incidence of airflow obstruction according to hemoglobin levels in men, women, and postmenopausal women

	Men (*N* = 1,594)		Women (*N* = 1,643)		Postmenopausal women (*N* = 1,055)	
	OR (95% CI)	*P*-value	OR (95% CI)	*P*-value	OR (95% CI)	*P*-value
Hb level, g/dL	0.83 (0.75, 0.92)	< 0.001	0.93 (0.74, 1.18)	0.568	0.82 (0.64, 1.06)	0.129
Age, years	1.06 (1.04, 1.08)	< 0.001	1.08 (1.05, 1.11)	< 0.001	1.08 (1.05, 1.12)	< 0.001
Smoking exposure, pack-years	1.01 (1.00, 1.02)	0.001	–	–	–	–
Area - Urban	0.51 (0.39, 0.66)	< 0.001	0.86 (0.54, 1.37)	0.531	0.95 (0.57, 1.60)	0.858
Menopause - Yes	–	–	0.62 (0.34, 1.13)	0.120	–	–

Abbreviations: CI, confidence interval; Hb, hemoglobin; OR, odds ratio

### Subgroup analyses in postmenopausal women

We conducted cross-sectional and longitudinal analyses in 1,507 postmenopausal women, and GEE analysis in 1,055 participants who underwent spirometry and Hb measurement at least four times. Among them, 78 women (7.4%) were diagnosed with AO during the follow-up period. Cross-sectional analysis (Table 2) showed a positive association between Hb levels and FEV_1_/FVC (estimate = 0.42%pred, *P* = 0.009). In the longitudinal analysis (Table 3), positive associations with both FVC (estimate = 11.8 mL, *P* < 0.001) and FEV_1_ (estimate = 9.8 mL, *P* < 0.001) were observed. In the GEE analysis (Table 4), Hb levels were not associated with the incidence of AO (OR = 0.82, *P =* 0.129).

## Discussion

In this study, we examined the impact of Hb levels on lung function and the incidence of AO in individuals from the general population. The cross-sectional analysis revealed a positive association between Hb levels and lung function. Using multiple linear mixed regression, we also examined the long-term association between Hb levels and lung function. In men, an increase in Hb level was associated with an overall improvement in lung function (FVC, FEV_1,_ and FEV_1_/FVC). However, in women, only FVC improved with Hb levels. In the GEE analysis, an increase in Hb levels was associated with a decreased risk of AO in men, but no significant changes were observed in women.

Several studies have shown a relationship between Hb levels and lung function in patients with COPD. An increase in Hb level is associated with lower disease severity, decreased comorbidities, lower mortality, and improved quality of life in these patients [11–15]. In addition, blood transfusion in patients with COPD facilitates ventilator weaning and reduces minute ventilation and work of breathing [26, 27]. The pathophysiology underlying these findings has not been clearly established; however, several theories have been proposed. First, as the Hb levels increase, the efficiency of pulmonary gaseous exchange is enhanced [28]. This implies that elevated Hb levels can contribute to improvements in exercise capacity. Second, blood transfusion can affect breathing patterns, potentially resulting in a reduction in minute ventilation and a decrease in the degree of hyperinflation [27].

An association between Hb levels and lung function has been observed in patients with sickle cell anemia [29–31]. While the mechanism by which Hb affects lung function in patients with sickle cell disease has not been fully elucidated, some studies suggest that patients with sickle cell anemia often have a lower dynamic lung volume, a higher incidence of restrictive spirometry, and decreased forced expiratory flow [29, 31, 32]. Moreover, these patients exhibit decreased respiratory muscle strength, which results in reduced lung volume [33, 34].

Few studies have analyzed the relationship between Hb levels and lung function in individuals from the general population [35, 36]; however, these studies were conducted with a small number of participants using cross-sectional analyses. In the present study, Hb levels were positively associated with long-term lung function decline and negatively associated with the incidence of AO. Further studies are required to investigate this mechanism, particularly in individuals from the general population.

We observed significant differences in baseline characteristics, including smoking exposure and menopausal status, between genders (Table 1). Therefore, we conducted separate analyses for men and women, which revealed that the impact of Hb on lung function and the OR of AO incidence varied by gender. Previous studies have reported that gender influences pulmonary function and the incidence of COPD [37–41], and the effect depends on anatomical, hormonal, social, economic, and cultural factors. Women generally have smaller respiratory systems, which could result in gender-based differences in lung function. Moreover, sex hormones can influence lung function by interacting with lung receptors, affecting airway tone and inflammation [38]. Smoking exposure and airborne occupational exposure contribute differently according to gender. Women are more susceptible to smoking exposure because their smaller lungs make them more vulnerable to the accumulation of harmful substances [40, 42]. In contrast, the effect of airborne occupational exposure on lung function decline was more pronounced in men. This difference is likely attributed to the composition of dust and the intensity of dust exposure [39]. These findings support the rationale for conducting separate statistical analyses for men and women.

Menstrual bleeding can cause fluctuations in the Hb level [24], and menopausal status may affect lung function [43, 44]. Therefore, we conducted a subgroup analysis including postmenopausal women. In postmenopausal women, Hb levels were positively associated with FEV_1_/FVC in the cross-sectional analysis and with FVC and FEV_1_ in the longitudinal analysis. However, the association with the OR of AO incidence was not significant.

Smoking exposure and area of residence were found to have significant associations with the OR for AO incidence in men. Smoking exposure, a well-established risk factor for AO [45], demonstrated a significant association with increased OR for AO incidence in our findings. This aligns with previous studies, highlighting the importance of addressing smoking as a modifiable risk factor in AO prevention strategies specifically for men. Regarding area of residence, significant differences in baseline characteristics were observed between urban and rural areas, which necessitated its inclusion as a covariate in our analysis. However, this study cannot conclusively determine whether area of residence directly influences AO incidence in men. Further investigation is warranted to explore the potential impact of urban-rural residence on AO development in men.

This study’s strength lies in examining the longitudinal association between Hb levels and lung function and the incidence of AO in individuals from the general population from a large-scale cohort study. Using serial follow-up data from the same participants, we determined the OR for AO incidence based on the Hb level.

However, our study has some limitations. First, it was an observational study; therefore, we could only discuss the association based on our study results rather than causation. Furthermore, the underlying pathophysiology remains unclear. Second, we did not consider comorbidities such as gastrointestinal bleeding, chronic kidney diseases, or hematologic disorders, which could affect Hb levels. In addition, we could not consider iron supplement intake in our analyses because the data were missing in many cases (41.1%). Even when iron supplementation data were available, the proportion of participants taking iron supplements was too low (0.4%) to be used for statistical analysis. However, we compensated for these limitations by excluding patients with abnormal baseline Hb levels. Third, lung function was not comprehensively assessed. We performed lung function assessments exclusively using pre-bronchodilator spirometry without including post-bronchodilator spirometry or imaging studies. In addition, even though it is crucial to consider exposure to air pollutants and symptoms for a more accurate diagnosis of COPD, we only used spirometry results. However, by conducting spirometry under strict quality control, we ensured the reliability of our lung function assessment. Fourth, emphysema patients are associated with hypoxemia and polycythemia, but we were unable to exclude them at baseline due to insufficient imaging data. We excluded all participants with airflow obstruction at baseline; however, as some emphysema patients can also have normal spirometry results, we could not exclude all emphysema patients.

## Conclusions

We investigated the long-term association between Hb levels and lung function in the general Asian population. Increased Hb levels were associated with lung function improvement. In addition, the incidence of AO decreased with increasing Hb levels in men. Therefore, a decrease in Hb levels should raise awareness about potential impairments in lung function.

## Electronic supplementary material

Below is the link to the electronic supplementary material.

Supplementary Material 1

## Data Availability

The data used in this study are publicly available from Korean National Institute of Health, Clinical and Omics Data Archive (CODA) at https://coda.nih.go.kr/.

## Abbreviations

AO: Airflow obstruction
CI: Confidence interval
COPD: Chronic obstructive pulmonary disease
FEV_1_: Forced expiratory volume in 1s
FVC: Forced vital capacity
GEE: Generalized estimating equation
Hb: Hemoglobin
OR: Odds ratio
WHO: World Health Organization
