# Supplementary material for: Longitudinal association between hemoglobin and lung function with insights into the incidence of airflow obstruction: an observational study
Source: BMC Pulm Med. 2025 Jan 30;25:50. doi: 10.1186/s12890-025-03505-3 (PMC11783840; doi:10.1186/s12890-025-03505-3)
Supplement: Supplementary file 1 — Supplementary Material 1 [file 12890_2025_3505_MOESM1_ESM.docx]

**Tables**

**Table S1** Number of participants in the Korean Genome and Epidemiology Study

|  | Year | From Ansan (urban) | From Ansung (rural) | Total participants | Participants in this study |
| --- | --- | --- | --- | --- | --- |
| Baseline | 2001-2002 | 5,012 | 5,018 | 10,030 |  |
| 1^st^ follow-up | 2003-2004 | 4,023 | 3,540 | 7,563 |  |
| 2^nd^ follow-up | 2005-2006 | 3,540 | 3,975 | 7,515 | 4,468 |
| 3^rd^ follow-up | 2007-2008 | 3,255 | 3,433 | 6,688 | 3,759 |
| 4^th^ follow-up | 2009-2010 | 3,262 | 3,403 | 6,665 | 3,707 |
| 5^th^ follow-up | 2011-2012 | 3,052 | 3,186 | 6,238 | 2,979 |
| 6^th^ follow-up | 2013-2014 | 3,000 | 2,906 | 5,906 | 3,324 |
